# Missed Opportunities in the Prevention of First Esophageal Variceal Bleeding: A Real-World Clinical Audit of Undiagnosed and Under-Treated Patients

**DOI:** 10.3390/jcm15176573

**Published:** 2026-08-26

**Authors:** Yusuf Bünyamin Ketenci, Hakan Demiröz, Mehmet Akca, İbrahim Gören, Ufuk Avcıoğlu, Ahmet Bektaş

**Affiliations:** Department of Gastroenterology, Ondokuz Mayıs University, Samsun 55270, Turkey; hakandemiroz.90@gmail.com (H.D.); md.makca@gmail.com (M.A.); drigoren@hotmail.com (İ.G.); ufukavcioglu@yahoo.com (U.A.); abektas@omu.edu.tr (A.B.)

**Keywords:** esophageal variceal bleeding, portal hypertension, splenomegaly, thrombocytopenia, primary prophylaxis, missed diagnosis, cirrhosis decompensation

## Abstract

**Background/Objectives:** Esophageal variceal bleeding (EVB) is a life-threatening complication of portal hypertension, yet many patients experience their first bleeding episode without prior diagnosis or adequate prophylaxis. This study aimed to describe the clinical, laboratory, and radiological findings present one year before the first EVB episode and to examine whether these findings were recognized and acted upon in clinical practice. **Methods:** We retrospectively reviewed 48 patients admitted with a first episode of EVB between January 2020 and January 2025. Demographic data, laboratory values, abdominal ultrasonography findings, and liver fibrosis scores were recorded both at the time of bleeding and during a baseline period of 12 ± 3 months prior to bleeding. **Results:** Of 48 patients, 22.9% (*n* = 11) had not been diagnosed with cirrhosis prior to bleeding, and 33.3% (*n* = 16) remained without primary prophylaxis despite a known diagnosis—meaning that 56.2% experienced their first EVB without effective preventive management. At baseline, splenomegaly was present in 90% of patients with available ultrasonography data, and 72.9% had platelet counts below 150 × 10^3^/µL. Annual changes in clinical scores (expressed as delta values) demonstrated progressive hepatic deterioration during the unmonitored period, as evidenced by worsening Child–Pugh and MELD scores. **Conclusions:** In the majority of patients who experienced a first EVB episode, simple and routinely available findings—namely splenomegaly and thrombocytopenia—were already present one year before the bleeding event. Furthermore, delta values indicate that disease severity progressively worsened during this unrecognized at-risk period. These findings represent missed clinical opportunities for earlier diagnosis and initiation of prophylaxis.

## 1. Introduction

Esophageal variceal bleeding (EVB) represents one of the most severe and life-threatening complications of portal hypertension, marking a critical transition from compensated to decompensated cirrhosis. Despite advances in endoscopic and pharmacological management, mortality rates following a first bleeding episode remain between 15 and 25%, underscoring the continued clinical burden of this condition [1,2,3].

Current guidelines, including the Baveno VII consensus, provide evidence-based recommendations for the screening and management of varices in patients with known cirrhosis or clinically significant portal hypertension. Non-invasive parameters, such as platelet counts below 150 × 10^3^/µL and liver stiffness measurements, are recommended to identify patients at risk and guide endoscopic screening [4,5,6]. When applied appropriately, primary prophylaxis with non-selective beta-blockers or endoscopic band ligation significantly reduces the risk of a first bleeding episode [2,4].

However, a clinically important and underexplored reality persists: a substantial proportion of patients present with their first EVB episode either without a prior diagnosis of cirrhosis or without having received adequate primary prophylaxis, despite being under medical follow-up [7]. In these patients, the transition to decompensation occurs silently, without a formal clinical response to warning signs that may already be present in routine evaluations. This represents not merely a disease progression phenomenon, but a potential missed opportunity in clinical care.

Simple, widely available findings—such as splenomegaly on abdominal ultrasonography and thrombocytopenia on a routine complete blood count—are well-established indirect markers of portal hypertension [3,8,9,10]. However, whether these findings are consistently recognized, documented, and acted upon in the period preceding a first bleeding episode remains poorly characterized in the literature. The majority of published studies have focused on the predictive value of instantaneous or static scoring systems at the time of clinical presentation, rather than on the clinical landscape in the months before decompensation [5,11,12,13].

The present study was designed to address this gap. We retrospectively examined the clinical, laboratory, and radiological findings available 1 year before the first EVB episode in a cohort of patients with portal hypertension, with the aim of characterizing the warning signs present during this pre-bleeding period and assessing whether they were recognized in clinical practice.

## 2. Methods

### 2.1. Study Design and Patient Selection

This was a single-center, retrospective observational study conducted at the Department of Gastroenterology, Ondokuz Mayıs University Faculty of Medicine, Samsun, Turkey. We retrospectively reviewed the records of patients admitted with a first episode of esophageal variceal bleeding (EVB) between January 2020 and January 2025. Ethical approval was obtained from the Ondokuz Mayıs University Ethics Committee (approval number 127/2025, dated 18 March 2025).

**Figure 1:**
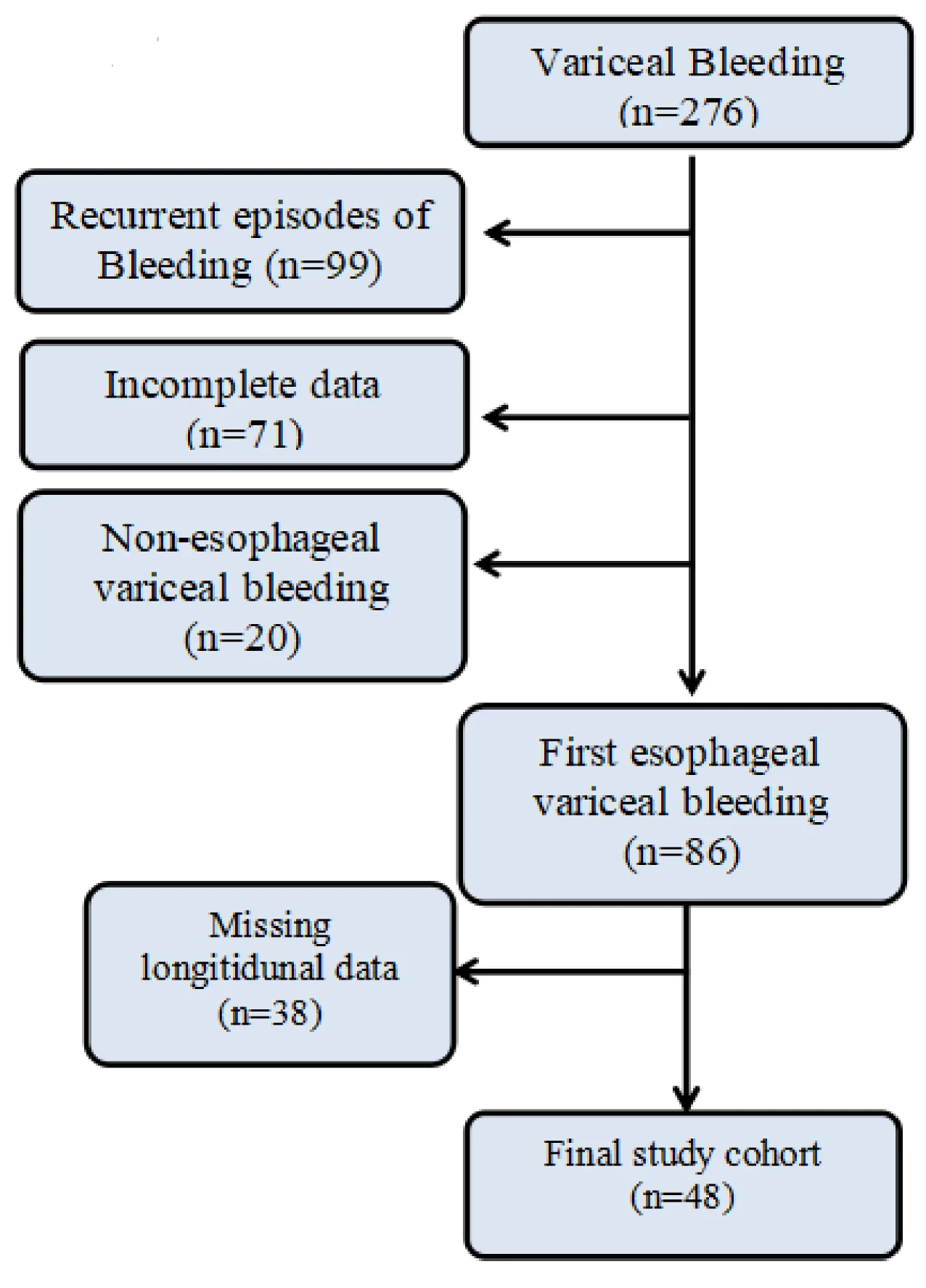
Flowchart of the study.

Patients were eligible for inclusion if they had a confirmed first episode of EVB, documented portal hypertension (cirrhotic or non-cirrhotic etiology), and available clinical, laboratory, and radiological records from both the day of bleeding and a baseline period of 12 ± 3 months prior to the bleeding episode. Patients were excluded if they had a prior history of variceal bleeding, extra-esophageal gastrointestinal variceal bleeding (gastric or duodenal), a concurrent diagnosis of hepatocellular carcinoma, or unavailable baseline data from the preceding year. Of the 86 patients initially identified, 38 were excluded due to missing baseline records, yielding a final cohort of 48 patients. Missing baseline data resulted mainly from incomplete outpatient follow-up before the bleeding event—a reflection of real-world gaps in continuity of care rather than systematic data loss (Figure 1).

To assess for selection bias, we compared baseline demographic and disease-severity characteristics between the excluded (*n* = 38) and included (*n* = 48) patients using the Mann–Whitney U test for continuous variables and Fisher’s exact test for categorical variables. No significant differences were observed in age, sex, etiology distribution, splenic diameter, platelet count, MELD, or Child–Pugh score at the time of bleeding (all *p* > 0.05, Supplementary Table S1), indicating that the excluded group was not systematically different from the analyzed cohort in baseline severity or demographic profile.

A subset of this cohort has been examined in a separate, related analysis addressing a distinct clinical question; no outcome variables, statistical comparisons, or tables are shared between the two manuscripts.

### 2.2. Data Collection

Demographic characteristics, etiology of liver disease, and prophylaxis status were recorded for all patients. Primary prophylaxis status was categorized as follows: patients with a confirmed diagnosis of cirrhosis or portal hypertension who were receiving non-selective beta-blockers at the time of bleeding were classified as ‘under prophylaxis’; patients with a confirmed diagnosis who were not receiving prophylaxis were classified as ‘diagnosed, not under prophylaxis’; and patients without a prior diagnosis of cirrhosis or portal hypertension were classified as ‘undiagnosed.’

Laboratory parameters—including complete blood count, liver function tests, and coagulation indices—and abdominal ultrasonography findings—including spleen diameter, portal vein diameter, and splenic vein diameter—were recorded for both time points. The upper limits of normal for ultrasonographic measurements were defined as: spleen diameter, 120 mm, portal vein diameter, 10 mm, and splenic vein diameter, 6 mm. Liver fibrosis and portal hypertension scores—including APRI, FIB-4, GPR, GAR, PALBI, AAPR, MELD, Child–Pugh (CTP), and PC/SD—were calculated at both time points for cirrhotic patients; PC/SD was additionally calculated for patients with non-cirrhotic portal hypertension. Annual changes in these scores were expressed as delta values (the value on the day of bleeding minus the value at baseline).

### 2.3. Statistical Analysis

Descriptive statistics were used to characterize the study population and the distribution of baseline clinical findings. The Wilcoxon signed-rank test was used to assess changes in paired parameters between the baseline and bleeding-day time points. All analyses were considered exploratory and hypothesis-generating. No multivariable adjustment was performed, given the limited number of outcome events. No formal correction for multiple comparisons (e.g., Bonferroni or false discovery rate control) was applied given the exploratory nature of the study and the limited sample size, which would make a conservative family-wise correction excessively restrictive. Consequently, all *p*-values in Table 1 and Table 2 should be interpreted as uncorrected and hypothesis-generating rather than confirmatory, and the reported delta values are presented as descriptive trends rather than validated predictors. All analyses were performed using SPSS version 22.0 (IBM Corp., Armonk, NY, USA).

## 3. Results

This study included 48 patients; 60.4% were male (*n* = 29) and 39.6% were female (*n* = 19), with a mean age of 61.33 ± 2.08 years. The most common etiology of liver disease was hepatitis B virus (HBV)-related cirrhosis (35.4%, *n* = 17), followed by metabolic dysfunction-associated steatotic liver disease (MASLD; 16.7%, *n* = 8), cryptogenic cirrhosis (14.6%, *n* = 7), autoimmune liver disease (12.5%, *n* = 6), alcohol-related liver disease (10.4%, *n* = 5), hepatitis C virus (HCV)-related cirrhosis (4.2%, *n* = 2), and non-cirrhotic portal hypertension (6.3%, *n* = 3).

At the time of the first bleeding episode, 43.8% of patients (*n* = 21) were under active primary prophylaxis with non-selective beta-blockers. Of the remaining patients, 33.3% (*n* = 16) had a known diagnosis of cirrhosis or portal hypertension but were not receiving prophylaxis, and 22.9% (*n* = 11) had not yet been diagnosed prior to the bleeding event. Consequently, 56.2% of patients experienced their first EVB episode without effective preventive management—either because they remained undiagnosed or because prophylaxis had not been initiated despite a known diagnosis.

When baseline ultrasonography data were examined, splenomegaly (spleen diameter >120 mm) was present in 90% of patients with available measurements (*n* = 27 of 30). Portal vein diameter exceeded the normal threshold in 62.1% of patients with available data (*n* = 18 of 29), and splenic vein diameter was enlarged in 44.8% (*n* = 13 of 29). Regarding platelet counts, 72.9% of patients (*n* = 35 of 48) had values below 150 × 10^3^/µL at baseline. These findings indicate that markers of portal hypertension—most notably splenomegaly and thrombocytopenia—were already present in the majority of patients one year before the first bleeding episode (Table 1).

Comparison of paired laboratory and radiological values between the baseline period and the day of bleeding demonstrated significant worsening across multiple parameters over the preceding year, consistent with progressive hepatic decompensation (Table 2).

Analysis of annual parameter changes (delta values) revealed continuous clinical deterioration in the 12-month period preceding the variceal bleed. Despite the presence of early warning signs, patients experienced progressive hepatic decompensation, as reflected by the worsening of median Child–Pugh scores (from 5.5 to 7; delta = 1) and MELD scores (from 8 to 12; delta = 3) between the baseline evaluation and the day of hemorrhage (Table 2).

## 4. Discussion

The present study retrospectively examined the clinical, laboratory, and radiological findings available one year before a first esophageal variceal bleeding episode in a cohort of 48 patients with portal hypertension. Our principal observation is straightforward: in the large majority of patients who experienced a first EVB episode, simple and routinely available markers of portal hypertension—most notably splenomegaly and thrombocytopenia—were already present one year before the bleeding event. However, more than half of these patients experienced their first bleeding episode without effective preventive management, either because they had not yet been diagnosed or because prophylaxis had not been initiated despite a known diagnosis. This pattern suggests that the clinical problem preceding a first EVB episode is not always the absence of warning signs, but rather the failure to recognize and act upon them.

This observation aligns with a growing body of evidence highlighting the gap between guideline recommendations and real-world clinical practice in the management of portal hypertension. The Baveno VII consensus criteria recommend initiating endoscopic screening and prophylaxis based on non-invasive parameters, including platelet count and liver stiffness measurement [4,5,6]. Our findings suggest that even the most basic of these parameters—platelet count and spleen diameter, both obtainable from a routine complete blood count and abdominal ultrasonography—were frequently abnormal in the year preceding a first bleeding episode, yet did not consistently trigger appropriate clinical action. We emphasize that, without a comparator group of stable compensated cirrhotic patients who did not bleed, we cannot determine whether the prevalence of splenomegaly and thrombocytopenia in this cohort exceeds their prevalence in the broader compensated-cirrhosis population; these findings are common decompensation-adjacent markers in general. Our claim is therefore narrower than ‘these findings predicted bleeding’: it is that, per Baveno VII criteria, platelet count <150 × 10^3^/µL and ultrasonographic splenomegaly should independently have triggered non-invasive risk stratification and consideration of screening/prophylaxis, irrespective of whether the individual patient was destined to bleed. The gap we describe is one of guideline-triggered action, not of bleeding prediction. Whether this reflects incomplete follow-up, inadequate risk stratification, or systemic barriers to care remains an important question that our retrospective design cannot fully address [8,9].

The subgroup of undiagnosed patients (22.9%) warrants particular attention. These patients had no prior diagnosis of cirrhosis or portal hypertension at the time of bleeding, yet their baseline ultrasonographic and laboratory findings were largely indistinguishable from those of the diagnosed subgroup. This underscores the well-recognized challenge of compensated cirrhosis, which may remain clinically silent until decompensation occurs [7]. In this context, opportunistic recognition of splenomegaly or thrombocytopenia during routine evaluations—even in the absence of a formal hepatological diagnosis—could represent a meaningful opportunity for earlier intervention [3,10,14]. Our finding that over half of the cohort (56.2%) experienced their first variceal bleeding episode without prior effective preventive management—despite the vast majority exhibiting splenomegaly (90%) and thrombocytopenia (72.9%) a full year prior—highlights a profound systemic issue of “clinical inertia”. In real-world healthcare workflows, patients frequently interface with primary care providers or general internal medicine clinics long before being referred to specialized gastroenterology or hepatology centers. In these non-specialized settings, indirect but classical markers of portal hypertension are often misinterpreted or insufficiently investigated. For instance, isolated thrombocytopenia may be erroneously attributed to primary hematological disorders (such as immune thrombocytopenia), while mild splenomegaly may be overlooked entirely, particularly if liver transaminases are within normal limits. This cognitive bias—failing to connect routine, readily available laboratory and radiological anomalies to underlying chronic liver disease in largely asymptomatic patients—prevents timely risk stratification. Consequently, the critical window of opportunity to initiate non-selective beta-blockers or endoscopic screening, as strongly advocated by the Baveno VII consensus, is missed. Ultimately, this gap between evidence-based guideline recommendations and everyday clinical implementation allows a preventable condition to transition silently into a life-threatening emergency. It should be noted that, without prior variceal grade or hepatic venous pressure gradient data, we cannot confirm that patients met formal Baveno VII criteria for primary prophylaxis specifically. What our data support is the narrower claim that these patients met non-invasive criteria (platelet count <150 × 10^3^/µL, splenomegaly) warranting endoscopic or elastography-based screening for clinically significant portal hypertension—the necessary preceding step to a prophylaxis decision, not a substitute for it.

The potential clinical relevance of dynamic score trajectories—rather than static values at a single time point—has been suggested in the prior literature in different clinical contexts [15,16]. However, given the methodological limitations of the present analysis, including the small event count, the absence of a comparator group, and the lack of external validation, these findings are strictly hypothesis-generating. We present them not as evidence of predictive utility but as observations that may motivate further investigation in larger prospective cohorts. The annual changes in prognostic scores (Delta-CTP and Delta-MELD) observed in our study quantitatively illustrate the “silent progression” of portal hypertension. These delta values confirm that patients did not decompensate suddenly; rather, their hepatic function deteriorated progressively over 12 months while they remained without adequate variceal screening or prophylaxis. This underscores the clinical inertia present in routine practice, where early warning signs fail to trigger necessary preventive algorithms.

We used the term ‘clinical inertia’ to summarize this pattern, but our retrospective design cannot distinguish among its possible drivers. These may include discontinuity between non-specialist and hepatology care, uneven access to liver stiffness measurement or timely ultrasonography outside referral centers, competing clinical priorities that deprioritize asymptomatic laboratory abnormalities, and—importantly—patients who do not seek care at all until a bleeding event occurs, which would appear identical to an in-system failure in a chart-based audit such as ours. We cannot adjudicate among these explanations with the present data, and future work should combine chart audit with structured assessment of referral timing, reasons for non-referral, and resource availability at the point of first contact. In particular, a dedicated prospective investigation—ideally combining structured chart audit with direct assessment of referral timing, resource availability, and patient-level barriers to care—is needed to disentangle the relative contributions of the candidate mechanisms outlined above (e.g., referral discontinuity, unequal access to elastography, workload-driven deprioritization, and delayed patient presentation). The present study can identify that a gap exists but cannot, by design, apportion it among these causes. We additionally note that even among the 21 patients receiving non-selective beta-blocker prophylaxis, endoscopic screening was not systematically documented, despite Baveno VII recommendations for endoscopic risk stratification and band ligation in high-risk varices—a further, specific illustration of guideline-practice divergence rather than a single unified phenomenon of ‘inertia.’

Several limitations of the present study must be acknowledged. The retrospective, single-center design limits the generalizability of our findings. No control group of cirrhotic patients who did not experience bleeding was included; therefore, no inference regarding the specificity of these findings for bleeding risk can be made. Because more than a dozen paired parameters were compared without adjustment for multiple testing, the nominally significant delta changes reported here carry an elevated risk of type I error and require confirmation in an independent, adequately powered cohort before any confirmatory interpretation.

This comparison addresses whether excluded patients differed in disease severity from the analyzed cohort, but it cannot address a more fundamental form of selection bias: patients with no baseline outpatient contact at all were excluded by definition because no baseline data existed to extract. This is precisely the subgroup most likely to represent complete diagnostic and preventive failure. Our cohort therefore describes missed opportunities among patients who had some prior engagement with the healthcare system; the 56.2% figure should not be generalized to all first-EVB presenters, and it likely, if anything, underestimates—rather than overestimates—the true population-level rate of missed prevention.

Despite these limitations, the central observation of this study carries practical relevance. The findings serve as a reminder that the clinical window preceding a first EVB episode is not always silent—it is often populated by recognizable, actionable findings that go unaddressed.

## 5. Conclusions

Esophageal variceal bleeding remains a preventable complication in a meaningful proportion of patients. The findings of this retrospective observational study demonstrate that, in the majority of patients who experienced a first EVB episode, recognizable markers of portal hypertension—particularly splenomegaly and thrombocytopenia—were already present one year before the bleeding event. However, more than half of these patients arrived at their first bleeding episode either undiagnosed or without effective prophylaxis, suggesting that the clinical challenge in this population lies not only in disease progression but also in the failure to translate available findings into timely clinical action.

These observations highlight the importance of systematic attention to routine, widely accessible parameters during the follow-up of patients at risk for portal hypertension decompensation. While the study’s retrospective design, small sample size, and absence of a comparator group preclude definitive conclusions, the central message is clinically actionable: simple findings available in routine practice may represent missed opportunities for earlier intervention.

Prospective, multicenter studies with standardized follow-up protocols are needed to confirm these observations and determine whether structured surveillance of non-invasive portal hypertension markers during the pre-bleeding period can reduce the incidence and mortality of first episodes of variceal bleeding.

## Figures and Tables

**Table 1:** Laboratory and radiological values on the day of bleeding, one year prior, and delta values.

Parameters	Median (Min-Max) One Year Ago	Median (Min-Max) Bleeding Day	DELTA (Min-Max)
Portal vein size (mm)	12.5 (10–17)	13 (6–19)	0 (−9, 5)
Splenic vein size (mm)	8 (6–20)	10 (4–20)	0.5 (−8, 8)
Platelet (10^3^/μL)	115 (45–385)	88 (42–516)	8.5 (−259, 248)
Spleen size (mm)	164.5 (120–250)	158.5 (114–223)	0 (−90, 41)

**Table 2:** Fibrotic and portal hypertensive scores on the day of bleeding, one year prior, and delta values.

Parameters	Median (Min-Max) One Year Ago	Median (Min-Max) Bleeding Day	Delta (Min-Max)
PC/SD (All cases)	724 (242.1–2549.2)	522.5 (214–3183)	−143.1 (−1216, 483)
MELD (Cirrhotic cases)	8 (6–23)	12 (6–39)	3 (−7, 22)
CTP (Cirrhotic cases)	5.5 (5–10)	7 (5–13)	1 (−2, 8)
Fib-4 (Cirrhotic cases)	4.4 (0.7–12.3)	5.7 (0.9–29.5)	0.7 (−7.2, 24)
APRI (Cirrhotic cases)	1.7 (0.3–9.1)	0.95 (0.1–2.6)	0.8 (−0.6, 7.9)
AARPRI (Cirrhotic cases)	2.3 (0.2–12.2)	3.6 (0.5–55.3)	1.2 (−5.4, 52.70)
GPR (Cirrhotic cases)	0.5 (0.1–2.7)	0.6 (0.1–23.9)	−1 (−2.4, 22.2)
GAR (Cirrhotic cases)	14.8 (4, 194.3)	15.5 (2.8–469.2)	0.3 (−49.7, 274.9)
PALBI (Cirrhotic cases)	31.6 (9.3–111.5)	28.25 (12.2–151.9)	0.45 (−91.9, 115)

## Data Availability

The data presented in this study are available upon request from the corresponding author. The data are not publicly available due to patient privacy and institutional confidentiality restrictions.

## Supplementary Materials

The following supporting information can be downloaded at: https://www.mdpi.com/article/10.3390/jcm15176573/s1, Table S1. Comparison of baseline demographic and disease-severity characteristics between included and excluded patients.
