# Supplementary material for: Missed Opportunities in the Prevention of First Esophageal Variceal Bleeding: A Real-World Clinical Audit of Undiagnosed and Under-Treated Patients
Source: J Clin Med. 2026 Aug 26;15(17):6573. doi: 10.3390/jcm15176573 (PMC13566761; doi:10.3390/jcm15176573)
Supplement: Supplementary file 1 [file jcm-15-06573-s001.zip › jcm-4475933-supplementary.pdf]

Supplementary Table S1. Comparison of baseline demographic and disease-severity characteristics between included and excluded patients.

Parameter	Included (n=48), mean	Excluded (n=38) mean	p value
**Age**	60,08	61,33	0.855[a]
**Sex, male,n(%)**	29 (60.4%)	25(%65.7)	0.658[b]
**Spleen/splenic diameter (mm)**	164,77	156,31	0.366[a]
**Platelet (10^3/µL)**	113,41	135,34	0.212[a]
**MELD**	15,29	14,20	0.685[a]
**CTP**	7,16	7,08	0.824[a]

a : Mann-Whitney test , b: Fisher Exact test
